# Rewriting the history of the invasive spiny water flea in North America

**DOI:** 10.1073/pnas.2622864123

**Published:** 2026-09-24

**Authors:** Danai Kontou, Philippa L. Ascough, Elizabeth J. Favot, Christopher Grooms, Andrew M. Paterson, John P. Smol, Andrew J. Tanentzap

**Affiliations:** ^a^ Ecosystems and Global Change Group, Department of Plant Sciences, University of Cambridge, Cambridge CB23EA, United Kingdom; ^b^ National Environmental Isotope Facility Radiocarbon Laboratory, Scottish Universities Environmental Research Centre, East Kilbride G750QF, United Kingdom; ^c^ Vale Living with Lakes Centre, Cooperative Freshwater Ecology Unit, Laurentian University, Sudbury, ON P3E2C6, Canada; ^d^ Paleoecological Environmental Assessment and Research Lab, Department of Biology, Queen’s University, Kingston, ON K7L3N6, Canada; ^e^ Dorset Environmental Science Centre, Environmental Monitoring Branch, Ministry of the Environment, Conservation and Parks, Dorset, ON P0A1E0, Canada; ^f^ Ecosystems and Global Change Group, School of Environment, Trent University, Peterborough, ON K9L0G2, Canada

**Keywords:** invasive species, global change, freshwaters, paleoecology, eDNA

## Abstract

Introduced species cause significant environmental impacts, making timely detection key for mitigation. The spiny water flea, *Bythotrephes longimanus*, is considered one of the most ecologically and economically destructive species in North American freshwaters. For decades, management focused on limiting its spread from the Laurentian Great Lakes, where it is believed to have been released via ballast water from trans-Atlantic ships entering the St. Lawrence Seaway in the 1980s. Combining microfossil analysis from ^210^Pb-dated sediments, radiocarbon dating of chitinous remains, and sediment DNA, we show *Bythotrephes* was present at low densities in inland lakes in Ontario, Canada, at least 100 years earlier than initially reported. We then consider how its recent proliferation coincided with widespread environmental change and shifts in other processes influencing its establishment and population growth. These results challenge widely accepted assumptions about the history of *Bythotrephes* in North America and highlight the importance of temporal perspectives for managing species introductions and their impacts.

## Significance

Introduced species cause major environmental harm, making timely detection essential for management. We present multiple independent lines of evidence that one of the most destructive species in North American freshwaters was established at least a century earlier than widely believed. These findings rewrite the history of an important invasive species and demonstrate the need to improve how we define and detect species introductions, especially because many species can persist at low densities for decades before population growth makes them easier to detect.

Biological invasions by introduced nonnative species are one of the largest threats to global biodiversity (1), with severe environmental and socioeconomic impacts (2). Although some species introductions are intentional, many are the indirect result of human activity, such as the removal of natural barriers (3) or the transportation of goods (4). For example, in the ecologically important Great Lakes—St. Lawrence River basin (hereafter Great Lakes basin), considered the world’s most invaded freshwater system, ~65% of alien species introductions have been attributed to the release of ballast water from transoceanic shipping (5). Species can also naturally expand their range due to environmental change or directly colonize new areas without any human interference (6), challenging our conception of indigeneity altogether (7). For example, the green iguana (*Iguana iguana*) spread unassisted across the Caribbean on logs and uprooted trees after the 1995 hurricane season (8).

Introduced nonnative species may be present in an area long before they become invasive, i.e., establish successive populations beyond their introduction point following active or passive spread (9). During this period, populations can persist beneath detection limits until a rapid increase in abundance is triggered by an environmental factor (10), continued propagule pressure (11), the introduction of favorable genotypes (12–14), and/or positive density dependence (15–17). This lag phase may offer the best opportunity to control population growth, limit geographic spread, and achieve complete eradication (18–20). However, determining the arrival time for a species into a region is notoriously difficult and can delay conservation action (4, 21). Given the longtimescales involved, we must often fit narratives to historical examples post hoc based on available evidence, introducing inevitable uncertainty with serious implications for management (18, 21). These uncertainties can lead to management strategies that prioritize preventing new introductions when invasion is instead driven by long-established but previously undetected populations responding to environmental change. For example, an Asian foraminiferan first reported in the eastern United States in 1961 was recently discovered in surrounding sediments dating to the early-19th century, suggesting past failed introductions and/or long-term cryptic presence (22), whereas its presence in Europe has recently been pushed back millennia with recent spread attributed to environmental change rather than introduction (23). Reconstructing species histories is therefore key to identifying the processes underlying population establishment and expansion, and thereby informing management, but remains challenging (21, 24–27).

The spiny water flea, *Bythotrephes longimanus* (Leydig 1860) (Cladocera: Cercopagididae)—hereafter *Bythotrephes*—is considered one of the most destructive invasive species in North American freshwaters (28). Once established, *Bythotrephes* can competitively displace native planktivorous invertebrates and fishes, restructuring entire food webs (29–33). Predation by *Bythotrephes* has reduced the biomass and diversity of native zooplankton by up to 34% and 54%, respectively, within lakes in the Great Lakes basin over the past 30 years (29–31). The virtual elimination by *Bythotrephes* predation of zooplankton grazers that control algal populations can further reduce water clarity. The cost of this ecosystem service degradation alone from the introduction of *Bythotrephes* is estimated at between US$87 and $163 million per lake (32).

**Fig. 1.**
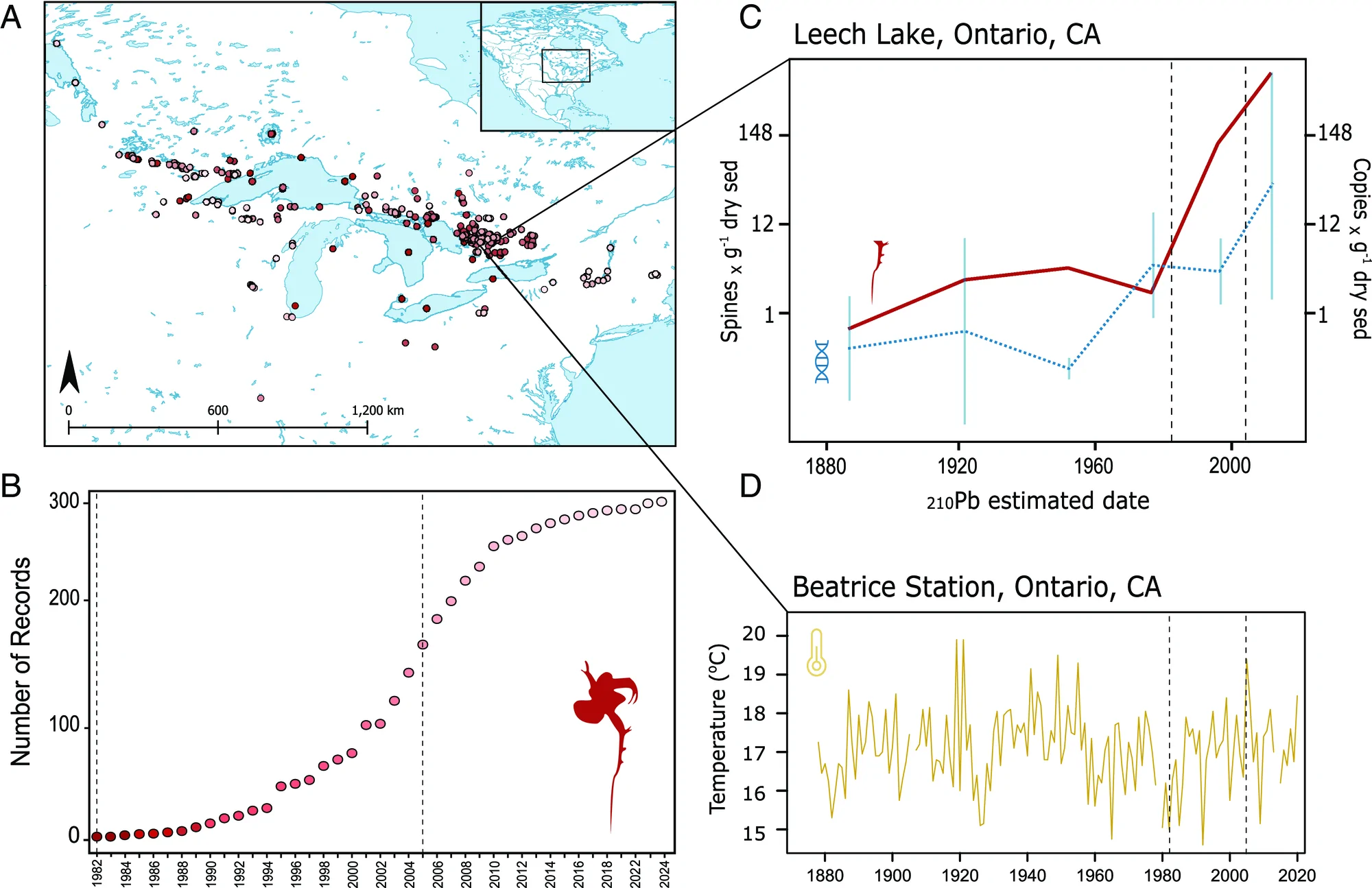
Establishment of *B. longimanus* across North America took >100 y and coincided with climate change. (*A*) We mapped the year that lakes were reported as first invaded by *Bythotrephes* based on direct observations in published databases and (*B*) plotted the cumulative number of invaded lakes over time. (*C*) In Leech Lake, Canada, we compared *Bythotrephes* spine densities (red line) with mean ± SE for copy numbers of a *Bythotrephes*-specific DNA marker amplified with quantitative PCR (blue line) in sediment (Pearson correlation between means: *r* = 0.99, *P* < 0.001, n = 6). (*D*) Mean air temperature in our study region (Muskoka, ON) between June and July, an important period for *Bythotrephes* reproduction. In all panels, vertical dashed lines mark the years 1982 and 2005 CE, which correspond to the first physical detection of *Bythotrephes* in the water column of Lake Ontario and Leech Lake, respectively.

*Bythotrephes* was long thought to have been introduced unintentionally from Europe into Lake Ontario from the ballast water of cargo ships in the early 1980s (31, 33–35). It was then presumed to have spread throughout the Great Lakes basin and across inland lakes of Canada and the United States (31, 36), with populations now reported in >300 lakes and rivers (Fig. 1*A*). Most water bodies were first identified as invaded between the late 1990s to early 2000s (Fig. 1*B*), but the total number is likely majorly underestimated. Most lakes are unmonitored, and identification relies on physical detection in the water column with plankton nets or fish stomach contents. Several million individuals may be required in a typical lake before *Bythotrephes* is detected by net sampling (37). Because *Bythotrephes* is primarily parthenogenic, that is, can reproduce by asexual clones alongside sexual reproduction, it can rapidly increase in number within a few months from as few as 10 eggs given environmental conditions that promote population growth (38). In its European range, *Bythotrephes* remained rare for decades in one long-term study lake, only increasing in density when growing seasons lengthened with warmer temperatures (39). Therefore, *Bythotrephes* could have been present and spreading undetected throughout North America for decades before reaching large enough populations to be reported in net samples. In support of this scenario, the characteristic chitinous spine and mandibles of *Bythotrephes* were recently detected in lake sediments from the United States and Canada that predated 1850 CE, thereby challenging the conventional introduction timeline (40). However, the lack of direct dating of these fossils, such as with radiocarbon, could not exclude the possibility that they were misattributed to older layers, such as from sediment mixing.

Determining when *Bythotrephes* arrived in North America is essential for understanding how different processes, including propagule pressure and environmental change, contributed to its spread and widespread detection in the late-20th century. Reportedly, some freshwater species, including invertebrates like the snail *Bithynia tentaculata* (41, 42), were introduced to the Great Lakes as early as the mid-19th century via trans-Atlantic vessels that carried solid ballast (41, 42). By the 1880s, these vessels could have also released ballast water into the Great Lakes (43–45). Once established, human activities could have transported *Bythotrephes* more widely (41, 42, 46) alongside endozoochory by fish (47) and waterfowl over short to moderate (<80 km) distances (48, 49). *Bythotrephes* specimens have been identified from freshwater ballast collected from the Great Lakes (31). However, repeat studies have not detected *Bythotrephes* individuals in ballast water or sediments from ships entering the Great Lakes (45, 50–52) and transiting European waters (53, 54), despite it being possible to do so with current methods (55). One explanation is that past studies have sampled only a minuscule fraction of the ballast water imported to North America, often collected in small subsamples, but *Bythotrephes* is also absent from freshwaters near the first point of introduction for trans-Atlantic cargo ships in Quebec and the Canadian Atlantic provinces according to species distribution data (56). The lack of records is notable because intensive ballast water inspection programs exist, and lakes in these regions have been extensively monitored with both traditional microscopy (57, 58) and environmental DNA (eDNA) (59, 60). Moreover, live transport of *Bythotrephes* is unlikely because individuals are known to have narrow physiological requirements when reared in the lab and are sensitive to injury and mortality from handling (61). Alternatively, resting eggs, which represent the more plausible vector for transport, cannot survive prolonged desiccation (>4 to 6 h) (62). Eggs would therefore have to be spread by water, such as from ballasts, but they are effectively absent in the water column of lakes with established populations (63) and in ballast water uptake by commercial ships in the Great Lakes (64). Furthermore, even if eggs reached the Great Lakes via trans-Atlantic vessels, no aquatic corridors connected these waters to small inland lakes before the development of canal and road networks in the mid-20th century. Although subsequent secondary dispersal inland cannot be excluded, such a pathway would require populations to be first established.

Here, we present multiple lines of evidence that *Bythotrephes* was already established in the Great Lakes basin at least a century before its first detection in 1982 in Lake Ontario (33), and likely much earlier. We then demonstrate that the increased abundance of *Bythotrephes* in recent decades has coincided with regional environmental change and explore the potential contributions from increased propagule pressure, genetic rescue and replacement, and the release of density-dependent demographic constraints. Our approach synthesizes counts of *Bythotrephes* remains and DNA detection with qPCR with direct radiocarbon (^14^C) dating of spine macrofossils. All these data were extracted from high-resolution ^210^Pb-dated sediment cores collected from seven lakes in south-central Ontario, Canada. Until recently, radiocarbon measurement from the chitinous exoskeleton of small invertebrates has been hindered by the ability to retrieve and purify a sufficient sample mass (65, 66), so the potential age of *Bythotrephes* remains has only ever been inferred from their stratigraphic position within sediment. Our work highlights that even the best-established narratives of species introductions can be incorrect in ways that have widespread consequences for ecosystem management.

## Results

### Detection of Exoskeletal Remains.

We consistently detected *Bythotrephes* chitinous caudal spines in the sediment of seven lakes in south-central Ontario, Canada, dating as far back as at least the late 19th century, and potentially earlier in two lakes (*SI Appendix*, Tables S1 and S2). These findings considerably predate the first observations of *Bythotrephes* from these lakes recorded between 2002 and 2006 in targeted plankton net samples, which supplemented monthly ice-free monitoring in the wider region since the early 1980s (36, 67). There were no caudal spines in sediment from four nearby lakes where *Bythotrephes* has never been observed in net samples (*SI Appendix*, Table S1). *Bythotrephes* spine densities consistently decreased in deeper sediments and ranged from 0.3 to 1,258 spines g^−1^ dry sediment (*SI Appendix*, Table S2). Chitin preserves exceptionally well for millennia even in harsh conditions (66), suggesting lower spine densities could be a true indication of lower abundances in the past (34, 68, 69). Extensive care was also taken to mitigate contamination and material transfer during sediment processing (*Materials and Methods*). Reconstructed profiles of chlorophyll-*a*, sedimentation rates, and radioisotope (^210^Pb, ^137^Cs, ^214^Bi) activities also did not reveal any signs of mixing that could influence stratigraphy of the sediment cores (*SI Appendix*, Figs. S1 and S2).

### Detection of Mitochondrial DNA.

In three of four invaded lakes where bulk sediment DNA was extracted, we detected *Bythotrephes* mitochondrial DNA at stratigraphic positions that predated the earliest observations from plankton net sampling but not the first appearance of spines in sediment. These sediments were ^210^Pb-dated to ~1988–2001 (Fletcher lake), ~1989–1998 (Grandview lake) and ~1867–1905 CE (Leech lake). In all three lakes, *Bythotrephes* DNA was consistently detected from these initial dates up to the present-day (*SI Appendix*, Fig. S4 and Table S3). In Leech lake, we further used qPCR results to quantify *Bythotrephes* abundance and observed a strong positive correlation between *Bythotrephes* DNA copies and spine density (Pearson correlation: *r* = 0.99, *P* < 0.001; Fig. 1*C*). Given the strong correlation with the density of exoskeletal material and the absence of distinct bioturbation signs in our cores (*SI Appendix*, Figs. S1 and S2), we consider DNA target detection in deeper sediments to be a true signal of past *Bythotrephes* presence and not an artifact of eDNA degradation or vertical migration within the profile (70, 71).

We never detected our *Bythotrephes* DNA target in the sediment of a lake that was presumed uninvaded based on plankton net tows (Bigwind lake) (36) or any contamination in negative (blank) controls. There was no detectable amplification in deeper sections of Fletcher and Grandview lakes or in any extracts from Otter lake, which was presumed invaded based on net tows from 2005 (*SI Appendix*, Fig. S4 and Table S3). Amplification failure in Fletcher and Grandview was likely due to low bulk DNA yields of 0.3 to 3.5 ng μL^−1^. Such low DNA yields, despite high spine densities, may be attributed to distinct sediment physicochemical properties that can significantly reduce eDNA retention and interfere with amplification downstream (72). Nevertheless, our results offer striking evidence of how targeted amplification of eDNA in sediment could complement monitoring efforts and help detect species of concern long before they are discovered using conventional techniques that often rely on high population densities.

### Radiocarbon Dating.

**Fig. 2.**
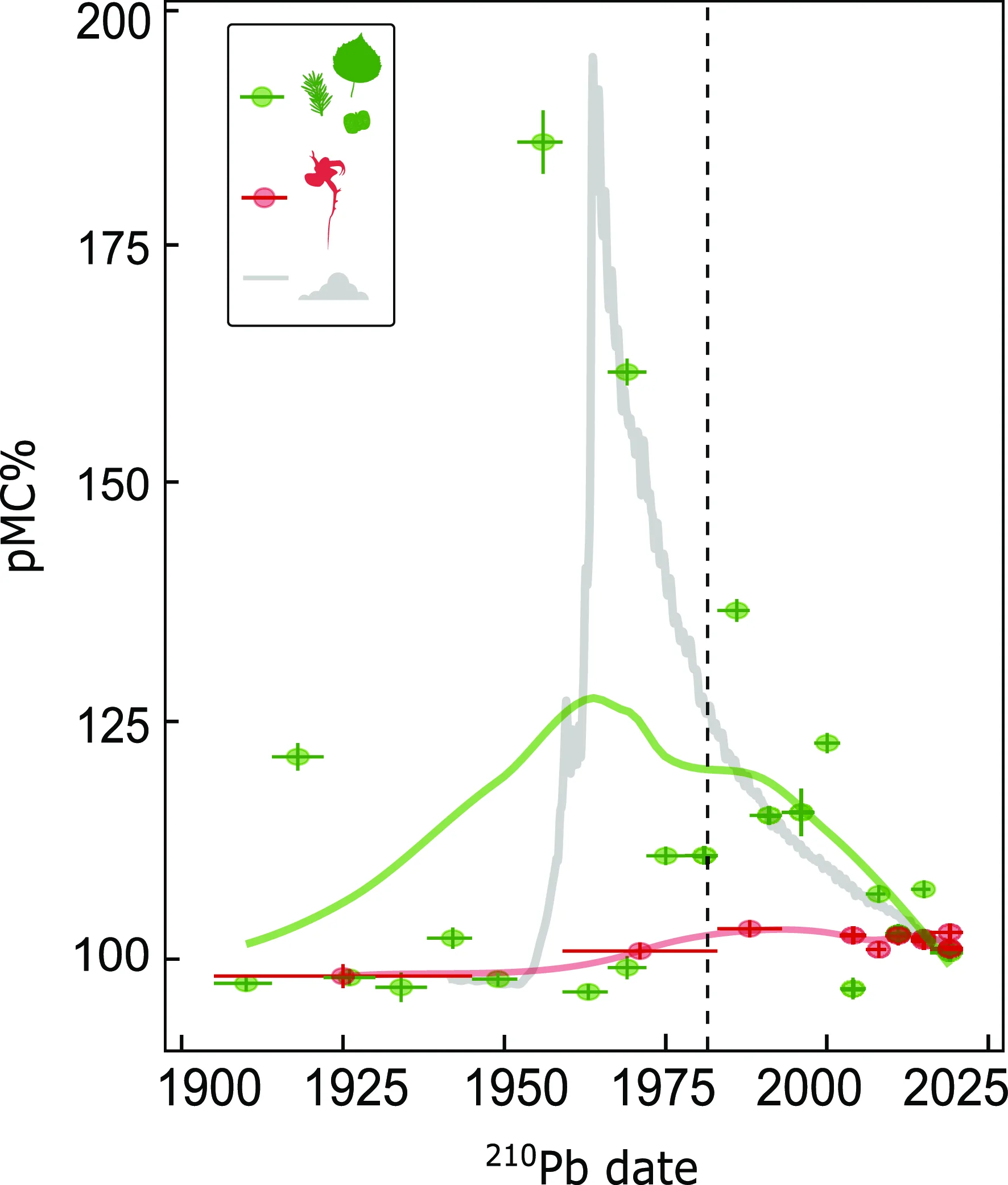
The spiny water flea was already established in Ontario, Canada by at least the 1950s according to multiple lines of radiometric dating. *B. longimanus* spine pools and terrestrial plant macrofossils (*Betula* spp. and *Picea* spp.) extracted from the sediment of Leech Lake, Ontario were radiocarbon dated. Radiocarbon results were expressed in percent modern carbon (pMC%) and plotted against lead (^210^Pb) dating of the sediment section from which the macrofossils were extracted. Sediment section dates in calendar years (CE) were inferred with a Constant Rate of Supply model and typically spanned periods of 5 to 10 years, so *x*-axis values are the estimated midpoint of each section. Bars on both axes represent 95% CI for each dating method. The gray line presents atmospheric ^14^C data for the temperate Northern Hemisphere zone 1. Coloured lines are smoothed splines fitted through respective observations. The dashed vertical line marks the first detection of *Bythotrephes* in net tow samples from Lake Ontario in 1982 and is widely accepted as the time of its establishment in North America.

In support of an earlier presence of *Bythotrephes* in North America, the oldest spine material extracted from the sediment of Leech Lake was estimated to have accumulated between 1905 and 1945 CE according to ^210^Pb dating (*SI Appendix*, Fig. S2 and Table S4). The same material was directly radiocarbon dated to a maximum possible calendar age of 1954 CE (*SI Appendix*, Table S4), as its ^14^C content was distinctly below 100% modern ^14^C (pMC) or <1 F^14^C (fraction modern ^14^C). This result indicates that the oldest set of spines in our study were formed before the bomb pulse of atmospheric radiocarbon in 1950 CE, in contrast to all other samples (>100 pMC) (*SI Appendix*, Fig. S2 and Table S4). We also radiocarbon-dated terrestrial plant material to provide another proxy for the age of our sediments (*SI Appendix*, Table S4). The pMC of individual *Betula* spp. seeds and leaf fragments, and *Picea* spp. needles, closely tracked atmospheric ^14^CO_2_ throughout the 20th century, further validating our aged stratigraphy (Fig. 2). The ^14^C measurements of spines from the youngest to oldest sediment sections similarly followed the rise and fall of atmospheric ^14^CO_2_, though these changes were more subdued (Fig. 2), as would be expected in a secondary consumer that obtains carbon through an aquatic food web (73). These results further suggest that the sediment cores had little physical mixing and *Bythotrephes* spines in the oldest section of our cores (9.5 to 10 cm depth) were likely already deposited in the sediment accumulated at the start of the 20th century.

Radiocarbon dates in freshwaters can appear older than their true age because carbon enters aquatic food webs from sources that are in disequilibrium with the atmosphere and thus depleted in Δ^14^C (74, 75). The most common source of this reservoir effect is dissolved inorganic carbon (DIC), which is fixed by photosynthetic organisms at the base of the food web and then makes present-day consumers appear artificially aged (75). However, there are reasons to exclude this process as an explanation for our results. First, this effect often originates from carbonate weathering of old bedrock or from export of carbon from deep soil layers into freshwater (75, 76). Both these scenarios are unlikely in our study lakes. Historical acid deposition has depleted calcium carbonate stores in the region (30), which were relatively negligible to start (77). The igneous geology of the Canadian Shield also limits soil development and groundwater, with most lakes dominated by surficial drainage. Therefore, DIC in our study lakes tends to exhibit modern radiocarbon ages (78) and calculations suggest that any potential contributions from weathering would be inconsequential (*Materials and Methods*). Second, *Bythotrephes* spine ages were not older than expected, and were within the anticipated age range based on ^210^Pb dating and the stratigraphy of where spines were found. Finally, we controlled for external contamination by aged carbon prior to dating.

## Discussion

By establishing a presence of *Bythotrephes* in the Great Lakes basin as far back as the 19th century, and likely longer (40), our findings challenge the established invasion history of this ecologically damaging species. The widely accepted timeline proposes *Bythotrephes* spread from a single founder population introduced from Eurasia into Lake Ontario within the last 40 years (31, 45, 79), but our study provides direct evidence that is incompatible with this timeline. The advance of our work was to radiocarbon-date macrofossil remains in ^210^Pb-dated sediments and amplify *Bythotrephes* mitochondrial DNA from this same sediment. This combination of methods allowed us to trace the temporal history of *Bythotrephes* with much greater confidence and precision. These results now raise important questions as to why it took decades to detect *Bythotrephes* in North America with broader lessons relevant for the study of species biogeography.

### An Earlier Presence in the Great Lakes Basin.

Determining if a species is introduced is extremely difficult because it relies on proving that a species did not exist somewhere previously, and the absence of evidence is not evidence of absence (24). In Ontario, Canada, for example, <1% of inland lakes are currently monitored, and even fewer are sampled for plankton. This data gap makes it easy to mistake a historically rare species that naturally colonized an area as newly introduced upon discovery. For example, if *Bythotrephes* was established in the Great Lakes basin before the Last Glacial Maximum, it would have undergone range contraction during glaciation and subsequently recolonized newly available habitats from refugia after glacial retreat ca. 10,000 to 15,000 years ago. This history would restrict *Bythotrephes* to isolated relic populations that easily evaded limited modern sampling until recent population increases made them noticeable. Alternatively, *Bythotrephes* may have arrived within the last 10,000 years via long-distance dispersal from Eurasia. Although long-distance dispersal is unlikely given *Bythotrephes*’ physiological constraints and low (<1%) viability after endozoochory (48), extremely rare events over evolutionary timescales cannot be excluded and likely explain the circumpolar distributions of many other invertebrate species (49, 80, 81). Millions of aquatic birds move between North America and Eurasia annually (82), and even a single *Bythotrephes* propagule can establish a population (83). Population genetic data to test this idea only place *Bythotrephes*’ arrival into North America sometime within the last 10,000 years (84). However, these data do show evidence of different postglacial expansion pathways that has resulted in genetic divergence between North American and European populations (84). These genetic differences could have resulted in traits that made North American populations historically rarer. For example, postglacial recolonization of other Cladocera show distinct genetic signatures from unglaciated populations, such as if they were associated with different dispersal and resource use strategies (80, 81). Importantly, whether *Bythotrephes* is native or introduced is far less important for management than understanding why a species has suddenly become invasive.

What is clear is that *Bythotrephes* was present in our study lakes at low densities for decades to centuries before its widespread detection. Introduced populations that exhibit these dynamics have been termed sleeper populations (10), and a prolonged lag phase before a species is detected at higher densities can give the impression that it was recently introduced. For example, the diatom *Didymosphenia geminata* remained undetected for centuries in Canadian waters until environmental change triggered the transition from rare to highly abundant populations that were presumed to be invasive (24, 85). Systematic monitoring of lake biodiversity in our study region only started in the 1980s, shortly before *Bythotrephes* was first recorded in the Great Lakes (31), so *Bythotrephes* likely occurred at densities well beneath detection thresholds of 0.5 individuals m^−3^ (37). In support of this explanation, sediment spine densities were relatively low until the 1970–1980s, and only rapidly increased from the 1990s to early 2000s (*SI Appendix*, Fig. S3), mirroring the exponential rise in first detection reports throughout North America (Fig. 1*B*). Discrepancies between first detection and the inferred timing of establishment have complicated the reconstruction of invasion histories in other species, requiring a similar integration of historical and genetic evidence (86).

### Explanations for Widespread Detection Since the 1980s.

*Bythotrephes* may have reached detectable densities in the Great Lakes during the early-1980s through processes that increased both propagule pressure and establishment success. The detection and rapid spread of *Bythotrephes* coincided temporally with many Eurasian freshwater introductions during the late-20th century, including highly invasive species such as *Gymnocephalus cernua* (ruffe), *Neogobius melanostomus* (round goby), *Dreissena polymorpha* (zebra mussel), and *D. bugensis* (quagga mussel) (44–46, 87). These introductions can be linked to elevated propagule pressure associated with intensified transoceanic shipping (44, 45). Higher propagule pressure, i.e., number and frequency of introductions, is well known to increase invasion success because repeated introductions of many individuals can increase the chances that some individuals survive, establish, and have the genetic variation to adapt to environmental differences (11). Strong density-dependent success of sexual reproduction and egg bank formation in *Bythotrephes*, i.e., Allee effects, would further increase population growth as more individuals established (31, 83). Taken together, these processes suggest that the timing of *Bythotrephes* detection in the early-1980s may be linked to the broader increase in propagule pressure across the Great Lakes rather than just a resurgence from a long cryptic residence.

Introductions of multiple genetic clades from distinct source populations may have also contributed to *Bythotrephes’* recent invasiveness. Ballast waters that carry relatively large numbers of propagules frequently result in admixed introduced populations that are better adapted at establishing across heterogeneous environments (13). Additionally, repeated introductions may provide genetic rescue that alleviates inbreeding depression and increases fitness independent of local adaptation (12, 88). For *Bythotrephes*, microsatellite analyses reveal evidence of initial founder effects that have potentially been offset by secondary introductions that increased genetic diversity (89), similar to observations from microsatellite analyses of invasive *D. bugensis* populations (90). These dynamics would mirror those of other taxa, such as in *Phragmites australis* (common reed), where introduced genotypes supplanted established lineages and enhanced invasion success with greater phenotypic plasticity and increased stress tolerance that enhanced growth rates (91). The heightened propagule pressure of the late-20th century would have accelerated both possibilities. However, it is worth repeating that *Bythotrephes* has never been detected in transoceanic ballast water, despite implementation of comprehensive binational inspection programs since the mid-2000s.

Environmental drivers may have further enabled *Bythotrephes* to proliferate. By analyzing long-term air temperatures from our study region, we found a unique confluence of two trends that coincided with the widespread detection of *Bythotrephes* in net samples across North America (Fig. 1 *A* and *B*), and that have been implicated in population growth of *Bythotrephes* in Europe and the United States (39). The first trend involves extreme cold summer temperatures followed by average or above-average summer temperatures (38, 92). During a historically cool summer in Wisconsin, Walsh et al. (83) found *Bythotrephes* massively increased investment in an egg bank, which stabilized the population at densities above detection limits after the eggs hatched when temperatures more closely resembled average conditions in the following year. In our study region, the summer of 1992, which corresponded to the start of the exponential rise in *Bythotrephes* detections (Fig. 1*B*), had the coldest recorded temperatures between 1878 and 2020, due to a global drop in temperature after the 1991 eruption of Mount Pinatubo (93) (Fig. 1*D*). Similarly, 1982 was the third-coldest summer in our study region, after which *Bythotrephes* was first detected in the Great Lakes (Fig. 1*D*). Both years were also much colder than expected simply by chance and followed by average years (*SI Appendix*, Fig. S5). Although many resting eggs can accumulate from parthenogenic reproduction and hatch under typical temperatures, field-based studies indicate that only large egg banks translate into increased population sizes because of excessive mortality from the resting egg to neonate life stage (94).

Once established at high densities, successively earlier growing seasons are likely another trend that has contributed to *Bythotrephes* population growth in the Great Lakes basin, similar to observations from Lake Maggiore, Italy (39). Summer temperatures, which are important for the annual development of plankton communities, increased by a mean ± SE of 0.07 ± 0.03 °C yr^−1^ between 1982, when *Bythotrephes* was first reported in the Great Lakes basin, and 2005, when it was first observed in three of our seven inland study lakes (linear model: *t*_22_ = 2.22, *P* = 0.037; Fig. 1 *B* and *D*). Together, high variability in summer temperatures between years, along with warmer average temperatures, could have triggered rapid population growth of *Bythotrephes*. This explanation matches observations for other introduced species (95) and forecasts for species range expansions and invasions facilitated by climate change (96).

Other environmental changes that arose after the 1970s likely had little consistent effect on *Bythotrephes* populations across North America. First, acidification of many North American lakes during the 1960s and 1970s (88–91) reduced dissolved organic carbon (DOC) concentrations and phytoplankton biomass (97, 98), the latter of which also arose from nutrient-control regulations after the 1972 Clean Water Act (99). These changes would together increase water clarity. Because *Bythotrephes* is a visual predator, favoring large clear water lakes in its native European range (100, 101), increased water clarity could have allowed it to survive better and establish across North America (35, 102, 103). However, both DOC and chlorophyll *a* concentrations have increased over this period in our study lakes (*SI Appendix*, Fig. S3) and the wider region (104), so water clarity is unlikely to be the primary explanation for increasing *Bythotrephes* populations. Second, introductions of invasive *Dreissena* mussels in the late-20th century have sharply increased water clarity across the Great Lakes and could have facilitated the success and spread of *Bythotrephes* consistent with the concept of an invasional meltdown, whereby early invaders facilitate the success of later arrivals (105, 106), but lake water calcium concentrations are too low in our study region to support mussels. Finally, in our study region (Muskoka, ON), historical acid deposition depleted lake calcium stocks and these effects have persisted to the present-day because of slow replenishment by mineral weathering (107). Experimental data show that *Bythotrephes* is largely insensitive to low calcium concentrations (108), while its calcium-dependent prey may experience limited growth rates and become increasingly vulnerable to predation, e.g., unable to produce physical defenses (109). Consequently, *Bythotrephes* may have more easily overcome prey defenses by the late-20th century, increasing foraging efficiency and subsequently growth and reproduction rates (110, 111). This explanation would, however, not apply to the Great Lakes and many other regions where calcium concentrations have remained nonlimiting to *Bythotrephes*’ prey (107).

### Conclusions: Implications for Ecology and Conservation.

The confirmation of a much earlier *Bythotrephes* presence than widely accepted offers insights into the colonization and spread of invasive species from sleeper and cryptic populations. A prolonged lag phase, especially at the centennial scale that we observed with *Bythotrephes*, could give species time to adapt and increase their potential to become invasive irrespective of whether they were introduced or native (18). Consequently, when populations are eventually detected, they might be so large and well-established that interventions may no longer be effective or feasible (10, 21). These findings have direct implications for how invasion histories can inform management. Rather than focusing on species-specific control, which can prove ineffective for invaders that have been established for centuries, we suggest managers consider ecosystem-based approaches that target the causes rather than symptoms of invasion (112). Frequent reintroductions, environmental change, and other introduced species may have all enabled the spread of *Bythotrephes*. Therefore, understanding the relative importance of factors that lead a species to become invasive may prove a more efficient use of resources than entirely removing established populations. Given the considerable funding allocated to biodiversity monitoring globally, we also emphasize the critical limitations of density-dependent detection methods and the benefits of adding historical context, such as from eDNA and paleoecology, to inform conservation and management. For example, targeting native species misidentified as introduced misdirects finite management resource away from genuine ecological threats. With the forecasted acceleration of global species movements (96, 113), there is an urgent need to improve monitoring strategies and timely detection of species introductions.

## Materials and Methods

### Long-Term Distribution and Climate Data.

Coordinates and year of first report of *Bythotrephes* were extracted from the United States Geological Survey Non-Indigenous Aquatic Species database v1.320 (accessed August 2024), the Global Biodiversity Information Facility (GBIF.org, accessed August 2024), the Canadian Aquatic Invasive Species Network technical report (36) and expert knowledge of additional studies not yet incorporated into these databases (35, 114, 115).

Historic temperature data for our study region (Muskoka–Haliburton, Ontario) were extracted from the Adjusted and Homogenized Canadian Climate Dataset from www.canada.ca/en/environment-climate-change/services/climate-change/science-research-data/climate-trends-variability/adjusted-homogenized-canadian-data.html (August 2024) for weather station: #6110607 in Beatrice, Muskoka, Ontario (Latitude: 45.16, Longitude: −79.39). Mean monthly temperatures for June and July were averaged for each year. We selected this period because it is known to affect *Bythotrephes* establishment, reproduction, and growth (38, 39, 92). For each year, we calculated the probability of a given temperature being observed using a Gaussian probability density function with mean and SD equal to the long-term (1880–2020) observations.

### Sediment Processing.

In autumn 2021, we collected four sediment cores (30 cm length; 7.6 cm diameter) from the deepest basin in each of seven lakes with known *Bythotrephes* presence since the early 2000s and four lakes with restricted public access and no recordings of *Bythotrephes*. The lakes were located across the Townships of Lake of Bays (Grandview, Longline, Clinto, and Otter lakes), Bracebridge (Leech, Bonnie and Bigwind lakes) and Haliburton County (Fletcher, Crown, Walker, and Buck lakes) in central Ontario, Canada (*SI Appendix*, Table S1). Cores were collected using a Glew gravity corer (116) and sectioned with a Glew extruder (117) at a resolution of 2 cm. One core from each lake was sectioned at a finer resolution of 0.5 cm for sediment analyses and dating with ^210^Pb and one core was set aside for DNA extractions. Sediment was immediately stored into sterile bags and tools were cleaned between sections with deionized water. In summer 2022, Leech lake was resampled for an additional 20 sediment cores (10 cm length; 7.6 cm diameter) sectioned at 0.5 cm to obtain sufficient material for ^14^C measurement. Each section was assessed in entirety to maximize material availability for radiocarbon dating, and extensive care was taken to avoid contamination by rinsing tools between depths. All samples were stored in the dark at ~4 °C until processing.

Sediment was processed for ^210^Pb gamma dating and spectral inference of chlorophyll-*a* concentration by the Paleoecological Environmental Assessment and Research Lab (PEARL), Queen’s University, Kingston, Canada. Dating followed Schelske et al. (118). Midpoint intervals from core sections were weighed, freeze-dried, ground, and stored in the dark for 14 d to achieve secular equilibrium of ^226^Ra and ^214^Bi. Radioisotope activities of ^210^Pb, ^137^Cs, and ^214^Bi were measured with an Ortec® high purity Germanium gamma spectrometer and core chronologies established according to a “Constant Rate of Supply” model (119). Dating results were analyzed using ScienTissiME (ScienTissiME Inc., Barry’s Bay, Canada). Chlorophyll-*a* (Chl*-a*) measurements followed methods described in Michelutti et al. (120). Similarly, DOC measurements followed Meyer-Jacob et al. (98). Sediment samples were lyophilized and sieved (125 μm) and then reflectance spectra (650 to 700 nm for Chl-*a*; 400 to 2,500 nm for DOC) were obtained with a spectroradiometer and calibrated using samples of known sedimentary concentrations (*SI Appendix*, Fig. S2).

### Isolation and Pretreatment of Spines and Plant Material.

The entire wet weight of individual sediment core intervals was filtered through metal sieves (150 μm) using deionized water starting from the deepest sections. Equipment was strictly separated for control and *Bythotrephes*-present lakes. Sieves were thoroughly rinsed and inspected under a stereomicroscope and gloves regularly changed to avoid cross-contamination and material transfer between sections. Filtrates were concentrated in sterile 50 mL tubes and stored in a dark room at 5 °C. We used sterile gridded petri dishes to examine all filtrates twice under a stereo microscope for *Bythotrephes* spines and plant macrofossils. Individual spines from all lake filtrates and identified plant material from the Leech Lake filtrates (*Betula* spp. seeds and leaf fragments, and *Picea* spp. needles) were counted and transferred to separate individual tubes with clean deionized water using sterile needles.

In preparation for ^14^C measurement, spines and a total of 10 representative plant samples recovered from Leech Lake cores were sequentially rinsed three times in ultrapure water with gentle agitation and examined under 100× magnification to confirm the removal of any physical contaminants. *Bythotrephes* spines required pooling to ensure accurate ^14^C measurement following chemical pretreatment due to their small size and weight (~10 mm; 64 μg per spine). Pooling of small invertebrate remains (e.g., insects) prior to dating is common practice for these reasons (66). Spines from multiple cores were therefore pooled in 12 batches of 62 to 143 spines. The grouping of spines from similar depths was guided by the ^210^Pb chronology and reconstructed chlorophyll-*a* profile. Use of both the ^210^Pb chronologies and reconstructed chlorophyll-*a* profiles, ensured accurate alignment across cores and the removal of any sections with detectable signs of potential disturbance, e.g., sediment mixing. Spine pools and seeds were air dried and transferred directly into sealed pretreated quartz tubes to avoid loss of material. Larger samples, such as leaves and needles, were sealed in sterilized glass vials for transport. In all cases, considerable measures were taken to eliminate contamination by external sources of aged carbon, including thorough removal of algal debris and chemical pretreatment of all samples prior to dating.

### DNA Extraction and Amplification.

We analyzed environmental (e)DNA from the sediments of four invaded lakes (Leech, Grandview, Fletcher, and Otter) using a quantitative (q)PCR assay originally developed by Walsh et al. (121) to detect *Bythotrephes* eDNA in lake water. We extracted bulk DNA from unfiltered lake sediment guided by the ^210^Pb dating profiles. In all cases, samples were selected to cover the period between the late 19th and early 21st century, extending well before and after the 1990–2000s when these lakes were first reported as invaded by *Bythotrephes*. As an additional control to identify potential nonspecific amplification, we also extracted DNA from the core top (0 to 2 cm) and bottom (18 to 20 cm) sediments of uninvaded Bigwind Lake, representing the time periods between 2010–2020 and 1884–1906, respectively.

We used ~250 mg of wet lake sediment per DNA extraction and each section was sampled in triplicate alongside extraction blanks with deionized water. Sediments from different lakes, core sections, and replicates were processed separately. We used a DNeasy PowerSoil® isolation kit (Qiagen, Hilden, Germany) and followed the manufacturer’s protocol for soil extractions. Extracted DNA was quantified with a high sensitivity DNA assay on a Qubit 3.0 fluorometer (ThermoFisher, Waltham, United States) and stored at −20 °C. Three replicate qPCR assays were prepared per extract using the Luna® Universal qPCR Master Mix (New England Biolabs, Ipswich, United States) and following the manufacturer’s recommended protocol for 20 μL reactions. We additionally prepared two sets of serial dilutions (1:10 up to 1:10^5^) for amplification using the top (0 to 2 cm) and bottom extracts (8 to 10 cm in Grandview and 10 to 12 cm in Leech lake) to test PCR efficiency and generate calibration curves for quantification. We targeted a conserved ~227 bp region of the mitochondrial cytochrome oxidase I (COI) for amplification using the *Bythotrephes* specific primers and thermal profile described in Walsh et al. (121). Sediment samples were accepted as positives for *Bythotrephes* if we observed consistent amplification of our target region in at least one of the replicate DNA extracts (*SI Appendix*, Fig. S4). Calibration curve intercepts and slopes were used to estimate target copy numbers from qPCR cycle threshold (Ct) values based on the standard equation: [(observed Ct) − (*y* intercept)]/slope. Copy numbers were then normalized and expressed as target copies per gram of dry sediment to allow for comparison with spine densities.

### Radiocarbon Dating.

Leech Lake samples were processed at the National Environmental Isotope Facility Radiocarbon Laboratory, Scottish Universities Environmental Research Centre, East Kilbride, UK. Plant samples (UCIAMS 281740-47, UCIAMS 281750-59, and UCIAMS 281762-64) were digested in 1 M HCl (80 °C, 2 h), washed free from mineral acid with deionized water, then digested in 0.5 M NaOH (80 °C, 2 h). Digestion was repeated using deionized water until no further humics were extracted. The residue was rinsed free of alkali, digested in 1 M HCl (80 °C, 1 h), then rinsed free of acid, and dried. Spine samples (UCIAMS 281732-36 and UCIAMS 284114, 284116-18) were digested in 1 M HCl at room temperature (21 °C, 2 h) while smaller spine pools from older sections (UCIAMS 289623-25) were digested in 1 M HCl at 80 °C (2 h). Samples were washed free from mineral acid with deionized water and dried. Total carbon of pretreated samples was recovered as CO_2_ by heating with CuO in sealed quartz tubes and the gas was converted to graphite by Fe/Zn reduction (122).

Accelerator Mass Spectrometry (AMS) analysis was performed at the Keck Carbon Cycle AMS Facility, University of California, Irvine, United States. ^13^C/^12^C ratio was measured by AMS during ^14^C determination and used to correct for isotopic fractionation after Stuiver and Polach (123), i.e., to normalize ^14^C to δ^13^C-VPDB‰ = −25. The data were corrected as described in Santos et al. (124) for modern carbon contamination and a correction based on the National Institute of Standards and Technology Oxalic acid II reference material. Radiocarbon dates were calibrated using the IntCal20 calibration curve for Northern Hemisphere Zone 1 for prebomb samples, and the Bomb NH1 curve for postbomb samples (125, 126). Calibrations were performed using the online tool OxCal v.4.4 (127).

Finally, we calculated the tolerance of our measurements to any potential contribution from aged DIC, and these suggest our dating results were robust to any effects from aged carbon reservoirs. If *Bythotrephes* were genuinely alive in our lakes between 1910 and 1914, atmospheric F^14^C would have averaged 0.994 (126). Assuming 5% of DIC used by phytoplankton was from geological weathering with F^14^C = 0 (128), which is similar to past studies (129), a simple mass balance would give phytoplankton a F^14^C = 0.945. Our own analyses of food web use, including in some of the same study lakes, found zooplankton grazers consumed by *Bythotrephes* consisted of 17 to 89% autochthonous material (130). The remainder of biomass derived from terrestrial organic matter that mirrors atmospheric ^14^C (130). Thus, algebraically solving a two-source mixing model for each scenario of minimum and maximum autochthonous resource use, the F^14^C of *Bythotrephes* from sediments ^210^Pb-dated from 1905 to 1914 should range between 0.986 and 0.951 respectively. This value is very similar to that observed of 0.982, further suggesting that any contribution of geological weathering to algae was small and even <5%.

## Supplementary Material

Appendix 01 (PDF)

## Data Availability

Supporting scripts and raw data are publicly available on Figshare (131). Study data and links to public datasets are included in the article and *SI Appendix*.
