# Supplementary material for: Rewriting the history of the invasive spiny water flea in North America
Source: Proc Natl Acad Sci U S A. 2026 Sep 24;123(39):e2622864123. doi: 10.1073/pnas.2622864123 (PMC13624641; doi:10.1073/pnas.2622864123)
Supplement: Supplementary file 1 [file pnas.2622864123.sapp.pdf]

PNAS

Supporting Information for

Rewriting the history of the invasive spiny water flea in North America.

Danai Kontou*, Philippa L. Ascough, Elizabeth J. Favot, Christopher Grooms, Andrew M. Paterson, John P. Smol, Andrew J. Tanentzap*

*Danai Kontou; Andrew J. Tanentzap

Emails: d.kontou@leeds.ac.uk (DK); atanentzap@trentu.ca (AJT)

This PDF file includes:

Figures S1 to S5

Tables S1 to S4

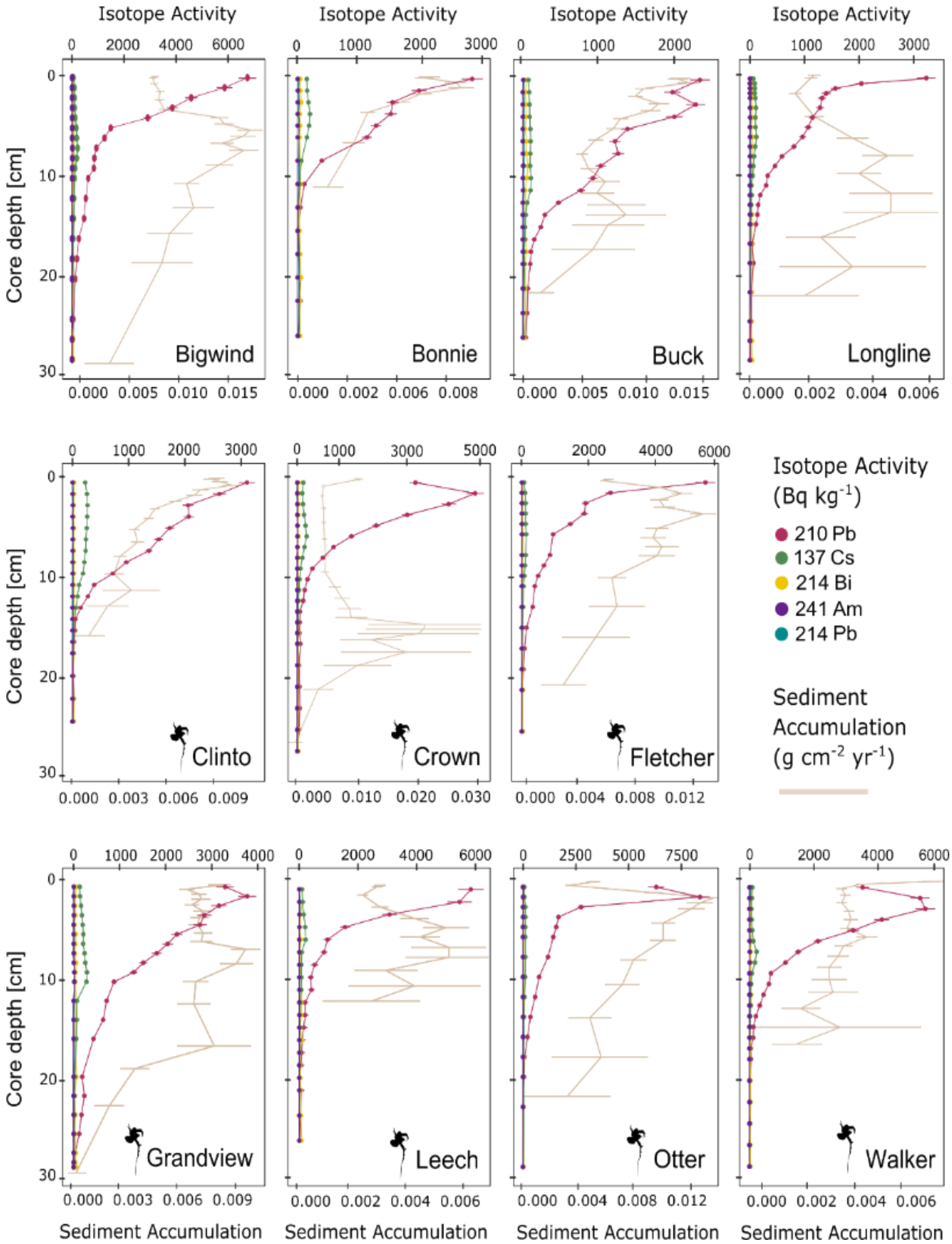

Figure S1. Isotope activity profiles, sediment accumulation rates and ^{210}Pb-inferred ages for the dated cores show no evidence of sediment mixing. Lines represent mean ^{210}Pb, ^{137}Cs, ^{214}Bi, ^{241}Am and ^{214}Pb total activity (Bq kg^{-1}) (top x-axis) and mean sediment accumulation (g cm^{-2} year) (bottom x-axis) plotted at core interval mid-points (0.5 cm) (y-axis). Error bars represent standard deviations, and x- and y-axis scales differ among plots. All cores were characterized by a steady decline in ^{210}Pb activity with depth, and low ^{137}Cs, ^{214}Bi, ^{241}Am and ^{214}Pb activities with minor stratigraphic variability and no evidence of major inversions or mixing. Interval dates for each lake were estimated according to the Constant Rate of Supply model up to 150 years before present.

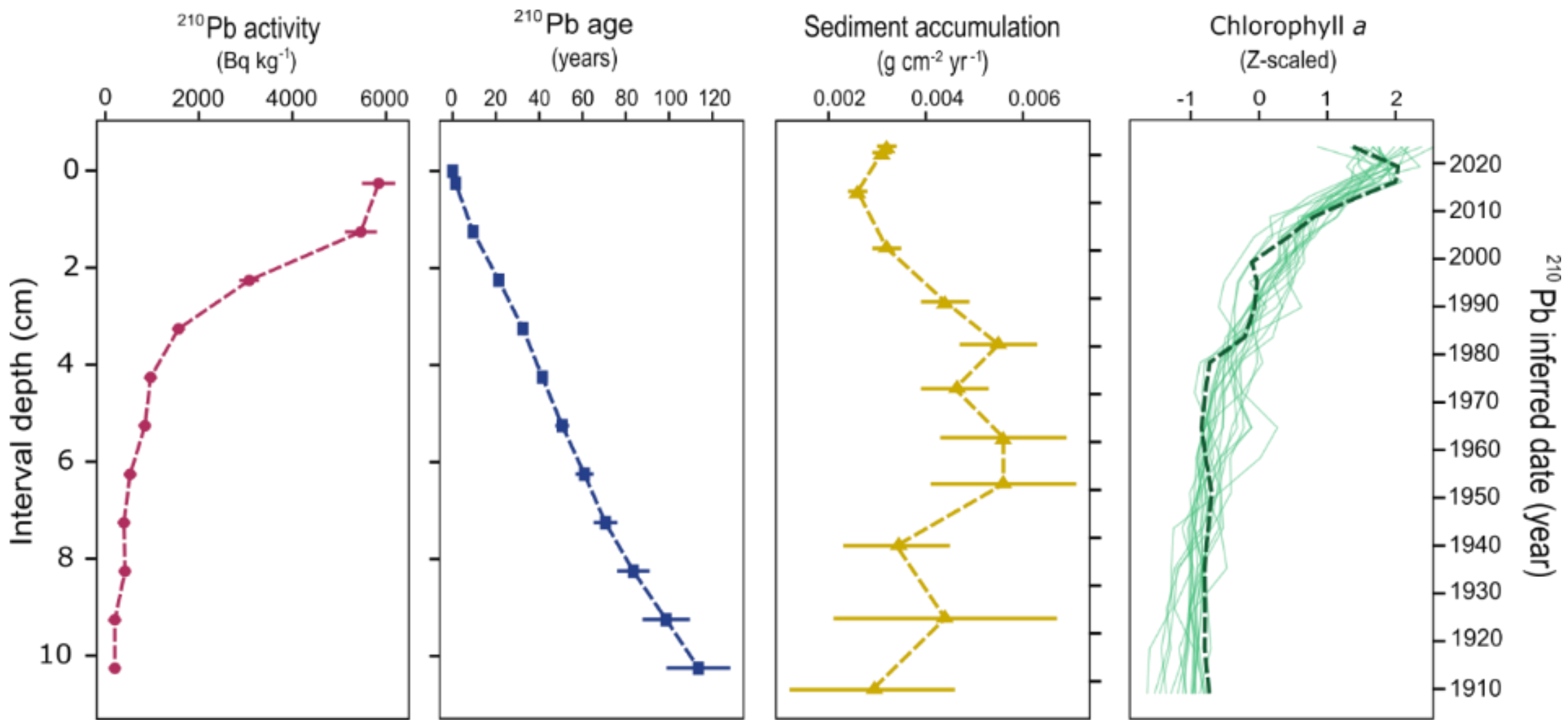

Figure S2. ^{210}Pb activity profile, inferred sediment age, sediment accumulation rates and reconstructed Chl-*a* profile comparison for replicate Leech Lake sediment cores show no evidence of sediment mixing and suggest close alignment. Dashed lines are profiles of the core used for 210Pb-dating, eDNA analyses and radiocarbon of plant macrofossils, whereas solid lines are additional cores used to ensure enough mass of *Bythotrephes* spines for radiocarbon dating. Chlorophyll-*a* values were scaled to a *Z*-score with a mean of zero and standard deviation of one so that their trends would be directly comparable and profiles could be aligned against the one core that was both ^{210}Pb-dated and analyzed for chlorophyll *a*. Lines in the first three panels represent mean ± standard deviation for ^{210}Pb total activity (Bq kg^{-1}), inferred age in calendar years before present, and mean sediment accumulation (g cm^{-2} year^{-1}) plotted at core interval mid-points (0.5 cm). Interval ages in calendar years were estimated according to a Constant Rate of Supply model. Green lines are chlorophyll-*a* profiles plotted at core interval mid-points (0.5 cm) reconstructed directly from sediment using visible spectroscopy.

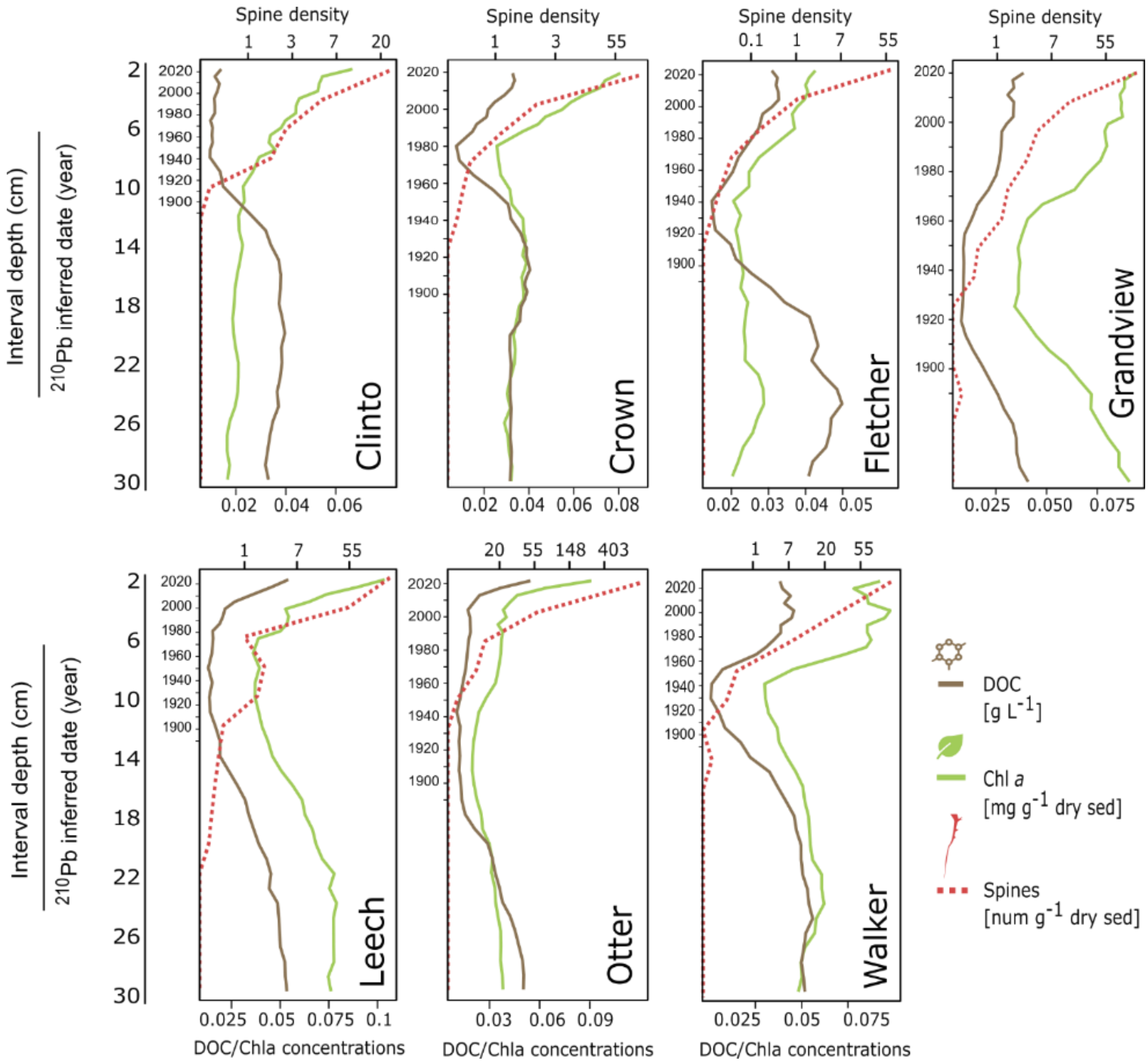

Figure S3. *Bythotrephes* spine densities increase following changes in lake water chemistry and clarity after lake acidification. Interval dates for each lake were estimated from ^{210}Pb activity based on the Constant Rate of Supply model up to 150 years before present and according to the profiles presented in Supplementary Fig. S1. Historical trends of chlorophyll *a* (Chl-*a*) and dissolved organic carbon (DOC) concentrations were reconstructed using visible spectroscopy from the same sediment cores. *Bythotrephes longimanus* caudal spine density (red) was plotted on a logarithmic scale for the same period.

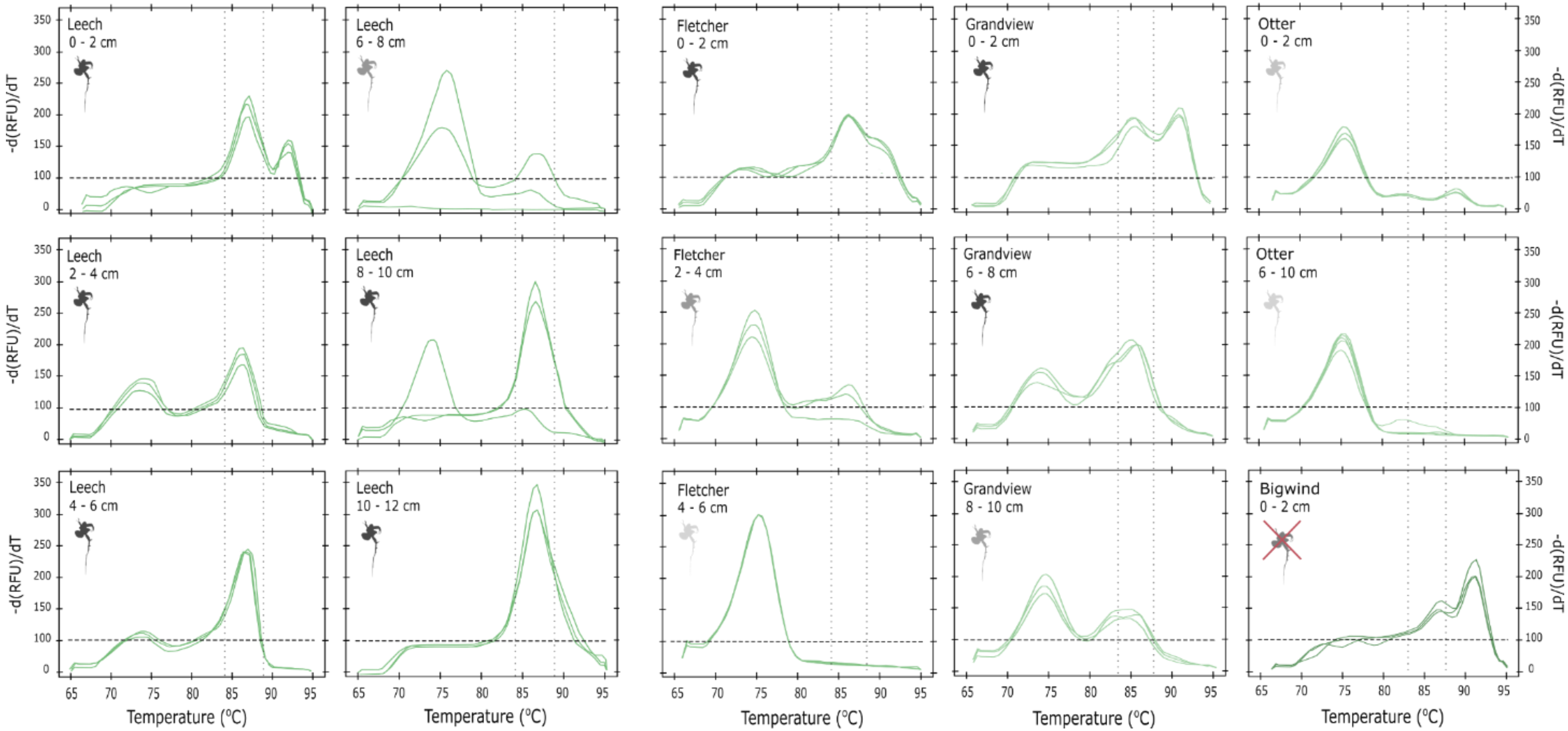

Figure S4. Examples of quantitative polymerase chain reaction (qPCR) melting curves with detection peaks for the sediment eDNA samples of three lakes invaded by *Bythotrephes* and an uninvaded negative control. The difference in fluorescence (relative fluorescence units – RFU) is plotted against melting temperature to detect DNA amplification peaks. Each solid green line represents one of three replicate DNA extract per section. Horizontal dashed line marks the detection threshold based on background fluorescence. Based on estimates from fragment size (~227 bp) and specific sequence GC content, the melting curve peak at ~86 °C (area between vertical dashed lines) corresponds to the target qPCR amplification product, a mitochondrial cytochrome oxidase I (COI) fragment, specific to *Bythotrephes longimanus*.

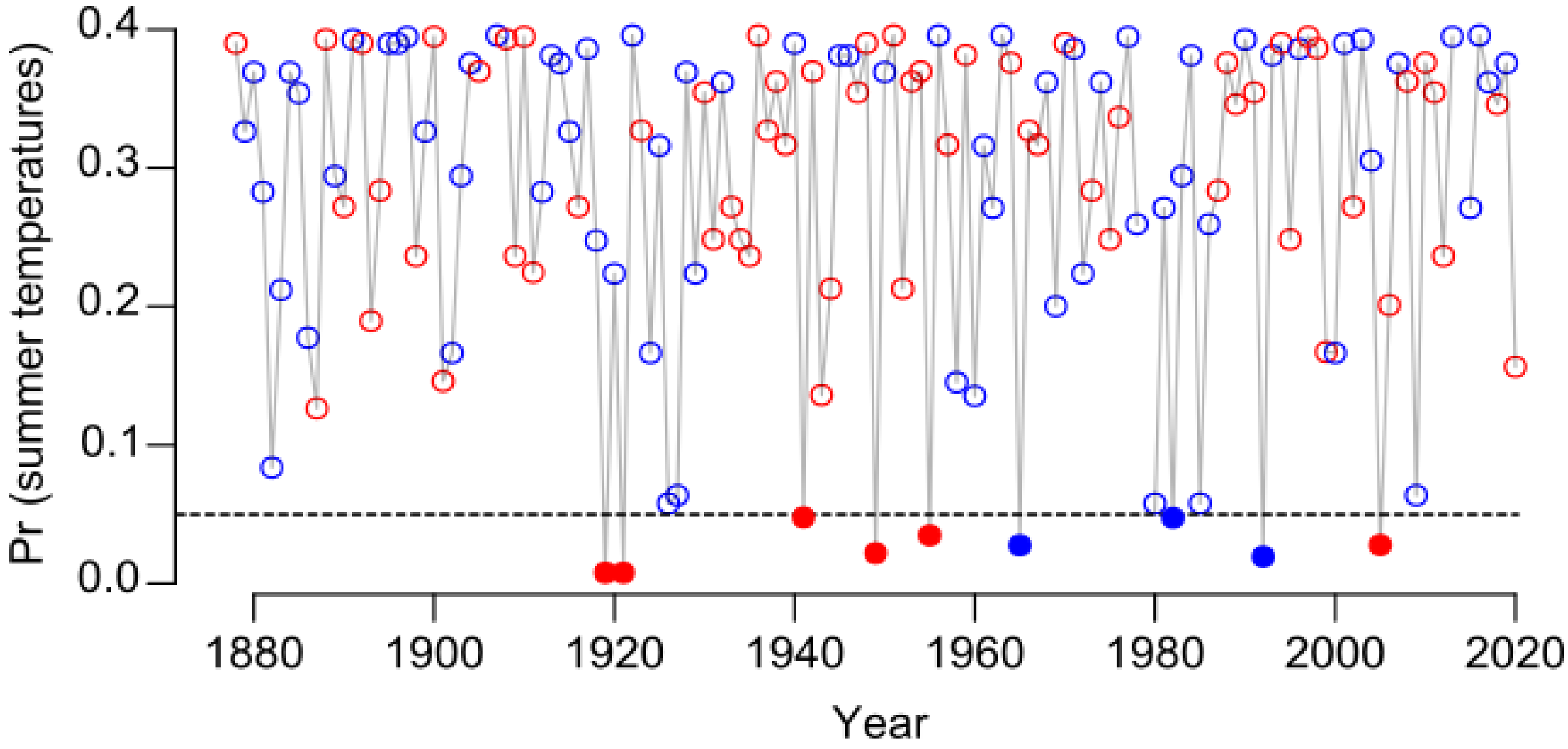

Figure S5. Probability of observing summer (June to July) temperatures in Ontario, Canada. Red and blue circles represent high and low temperatures, respectively, compared to the long-term mean from 1880 to 2020. Nine years within the last century (filled circles) recorded temperatures far above or below the long-term mean, i.e. probability of being observed <0.05. The probability of observing each temperature in a given year was calculated using a Gaussian probability density function with mean and standard deviation equal to the long-term values. Historical climate data were extracted from the Adjusted and Homogenized Canadian Climate Dataset (www.climate-change.canada.ca, accessed Aug 2024) for weather station: #6110607 (Beatrice, Ontario, Canada, Latitude: 45.1585, Longitude: -79.3827).

Table S1. Sampling information for 11 study lakes in Ontario, Canada. Accessibility, shoreline development and earliest *Bythotrephes longimanus* detection records provided by the Ontario Ministry of Natural Resources and Forestry (2010; 2021). Asterisk (*) denotes year of first detection in the water column.

Lake	Latitude	Longitude	Surface (ha)	Elevation (masl)	Max depth (m)	Dates sampled	Lake access	*Bythotrephes*
Bigwind	450570	-790540	108.0	335.0	36.0	22/10/2021	Public	No
Bonnie	450800	-791600	41.8	297.0	22.0	21/10/2021	Private	No
Buck	453914	-789910	40.0	378.0	27.0	09/11/2021	Private	No
Longline	451500	-785900	27.1	336.0	16.8	15/10/2021	Private	No
Clinto	451900	-785200	137.6	425.0	21.3	13/10/2021	Public	Yes (2005*)
Crown	452600	-784000	136.0	480.0	32.0	14/10/2021	Public	Yes (2006*)
Fletcher	452100	-784700	255.8	410.0	23.2	20/10/2021	Public	Yes (2006*)
Grandview	451200	-790300	76.9	345.0	22.9	15/10/2021	Public	Yes (2002*)
Otter	452766	-788752	116.6	378.0	19.5	13/10/2021	Public	Yes (2005*)
Walker	452400	-790500	68.2	361.0	17.0	12/10/2021	Public	Yes (2003*)
Leech	450500	-791000	81.0	309.0	13.0	21/10/2021<br>27/07/2022	Public	Yes (2005*)

Table S2. ***Bythotrephes* spine densities in invaded lakes**. Densities were expressed as number of spines extracted per gram of dry sediment. Core depth corresponds to stratigraphic position. The first appearance of *Bythotrephes* in the sediment record of each lake is bolded. The ^{210}Pb-inferred date range (CE years) of the sediment (bottom line) is reported for the corresponding depth in which *Bythotrephes* was first detected. Asterisk (*) denotes ages > 150 years and past the dating limit for ^{210}Pb isotopes.

Core depth (cm)	Leech	Fletcher	Grandview	Otter	Clinto	Crown	Walker
**2**	666.7	275.6	424.9	1258.0	41.9	269.3	58.5
**4**	99.0	17.8	24.1	44.1	8.6	8.0	10.5
**6**	1.7	10.1	8.0	8.4	3.6	2.1	3.5
**8**	3.4	2.8	5.2	12.4	2.4	0.7	1.1
**10**	2.5	1.0	2.5	5.9	**0.6**	0.5	0.8
**12**	0.6	1.2	2.8	0.7	0.0	**0.4**	0.0
**14**	0.5	1.5	1.1	0.6	0.0	0.0	**0.5**
**16**	0.5	0.0	0.9	**0.5**	0.0	0.0	0.0
**18**	0.3	0.4	0.0	0.0	0.0	0.0	0.0
**20**	**0.5**	0.3	0.0	0.0	0.0	0.0	0.0
**22**	0.0	0.3	0.0	0.0	0.0	0.0	0.0
**24**	0.0	0.0	**0.5**	0.0	0.0	0.0	0.0
**26**	0.0	**0.5**	0.0	0.0	0.0	0.0	0.0
**28**	0.0	0.0	0.0	0.0	0.0	0.0	0.0
**30**	0.0	0.0	0.0	0.0	0.0	0.0	0.0
**^{210}Pb date (AD)**	pre-1870*****	pre-1870*****	1887-1906	1897-1924	1912-1951	1922-1938	1866-1900

Table S3. Comparison of *Bythotrephes* spine sediment densities with mitochondrial DNA target copy numbers from a quantitative polymerase chain reaction (qPCR). Core section depths correspond to stratigraphic position and cover the period between late 19th and early 21st Century. Crosses denote the number of replicate DNA extracts from 1 to 3 with successful amplification. Cross in parentheses (+) marks a single extract with amplification below the quantification threshold.

Lake	Core depth (cm)	^{210}Pb date range (CE)	Spines / g dry sed	Copies / g dry sed	Detection peaks
	0 - 2	2006-2021	666.67	581.97	+++
	2 - 4	1988-2006	99.01	58.08	+++
Leech	4 - 6	1966-1988	1.74	84.72	+++
	6 - 8	1938-1966	3.39	5.86	++
	8 - 10	1905-1938	2.46	19.66	+
	10 - 12	1867-1905	0.65	13.91	+++
	0 - 2	2017-2021	424.93	10.64	+++
	2 - 4	2012-2017	24.11	27.47	+++
	4 - 6	2005-2012	8.03	-	(+)
	6 - 8	1998-2005	5.23	0.49	+++
	8 - 10	1989-1998	2.47	1.59	+++
Grandview	10 - 12	1978-1989	2.81	0.00	
	12 - 14	1967-1978	1.12	0.00	
	14 - 16	1953-1967	0.92	0.00	
	16 - 18	1939-1953	0.00	0.00	
	18 - 20	1923-1939	0.00	0.00	
	20 - 22	1906-1923	0.00	0.00	
	22 - 24	1887-1906	0.50	0.00	
	0 - 2	2011-2021	275.63	74.22	+++
	2 - 4	2001-2011	17.84	13.79	++
	4 - 6	1988-2001	10.15	-	(+)
Fletcher	6 - 8	1971-1988	2.83	0.00	
	8 - 10	1952-1971	0.99	0.00	
	10 - 12	1929-1952	1.19	0.00	
	12 - 14	1903-1929	1.48	0.00	
	14 - 16	1874-1903	0.00	0.00	

Table S4. Comparison of ^{210}Pb and ^{14}C dating results for *Bythotrephes* spine pools and individual plant samples from the sediment of Leech Lake, Ontario, Canada. Sample codes are uniquely assigned lab identifiers. Stratigraphic position corresponds to sediment core depth. ^{210}Pb dates are listed as calendar years (CE). $F^{14}C$ refers to fraction modern ^{14}C and ''1σ unc.'' to dating uncertainty at one standard deviation (68%). The last four columns present the upper and lower limits of calibrated date ranges in calendar years (CE) at 2σ (95%) dating uncertainty. Where the upper limit is the year of sampling (2021), date is given in parentheses. For samples that predate the bomb peak, the upper limit is marked with an asterisk (1950*). Pre-bomb dates (pre-1950) were calibrated for Northern Hemisphere Zone 1 using the IntCal20 calibration curve and post-bomb dates (post-1950) using the Bomb21 curve. All dates were calibrated using the OxCal v.4.4 online interface.

**Sample code**	**Type (Quantity)**	**Strati-graphic position (cm)**	**^{210}Pb date (CE)**		**$F^{14}C$**	**1σ unc.**	**2σ date range (CE)**			
							**older**		**younger**	
			**Lower**	**Upper**			**Lower**	**Upper**	**Lower**	**Upper**
UCIAMS-281732	*Bythotrephes* spines (143)	0.0-0.5	2016	2021	1.0117	0.004	1955	1956	2016	(2021)
UCIAMS-281733	*Bythotrephes* spines (143)	0.0-0.5	2016	2022	1.0283	0.005	1956	1956	2012	2017
UCIAMS-281734	*Bythotrephes* spines (143)	0.0-0.5	2016	2023	1.0106	0.004	1955	1956	2016	(2021)
UCIAMS-284114	*Bythotrephes* spines (137)	0.5-1.0	2013	2016	1.0224	0.005	1955	1956	2013	2018
UCIAMS-284116	*Bythotrephes* spines (136)	0.5-1.0	2013	2017	1.0196	0.004	1955	1956	2014	2019
UCIAMS-284117	*Bythotrephes* spines (108)	1.0-1.5	2009	2013	1.025	0.005	1955	1956	2013	2018
UCIAMS-284118	*Bythotrephes* spines (107)	1.0-1.5	2009	2013	1.026	0.006	1955	1956	2013	2018
UCIAMS-281735	*Bythotrephes* spines (119)	1.5-2.0	2006	2009	1.0103	0.004	1955	1956	2016	(2021)
UCIAMS-281736	*Bythotrephes* spines (83)	2.0-2.5	2002	2006	1.0251	0.005	1955	1956	2013	2018
UCIAMS-289623	*Bythotrephes* spines (114)	3.0-4.5	1983	1993	1.0322	0.005	1956	1957	2011	2016
UCIAMS-289624	*Bythotrephes* spines (90)	4.5-6.5	1959	1983	1.0088	0.004	1955	1956	2017	(2021)
UCIAMS-289625	*Bythotrephes* spines (62)	7.5-10	1905	1945	0.9824	0.006	-	-	1667	1950*
UCIAMS-281740	*Picea* sp. needle (1)	0.0-0.5	2016	2021	1.0067	0.004	1955	1956	2017	(2021)
UCIAMS-281741	*Betula* sp. seed (1)	0.5-1.0	2013	2016	1.0736	0.005	1957	1958	2002	2007
UCIAMS-281742	*Picea* sp. needle (1)	1.0-1.5	2009	2013	1.0271	0.005	1956	1956	2013	2017
UCIAMS-281743	*Picea* sp. needle (1)	1.5-2.0	2006	2009	1.0686	0.005	1957	1957	2003	2007
UCIAMS-281744	*Betula* sp. seed (1)	2.0-2.5	2002	2006	0.9693	0.006	-	-	1493	1950*
UCIAMS-281745	*Betula* sp. seed (1)	2.5-3.0	1998	2002	1.2271	0.005	1959	1962	1983	1985
UCIAMS-281746	*Betula* sp. seed (1)	3.0-3.5	1993	1998	1.1543	0.013	1958	1959	1988	1994

UCIAMS-281762	*Betula* sp. leaf (1)	3.5-4.0	1988	1993	1.1087	0.005	1958	1959	1990	1993
UCIAMS-281763	*Betula* sp. leaf (1)	4.0-4.5	1983	1988	1.6167	0.007	1962	1962	1976	1977
UCIAMS-281764	*Betula* sp. leaf (1)	4.5-5.0	1978	1983	0.9658	0.004	1958	1958	1995	1999
UCIAMS-281747	*Betula* sp. leaf (1)	5.0-5.5	1972	1978	1.8582	0.017	1958	1959	1995	2000
UCIAMS-281750	*Betula* sp. leaf (1)	5.5-6.0	1966	1972	0.9793	0.004	1963	1963	1968	1968
UCIAMS-286728	*Betula* sp. seed (1)	5.5-6.0	1966	1972	0.9913	0.006	-	-	1680	1950*
UCIAMS-281751	*Picea* sp. needle (1)	6.0-6.5	1959	1966	1.0222	0.006	-	-	1494	1950*
UCIAMS-281752	*Betula* sp. seed (1)	6.5-7.0	1952	1959	0.9707	0.008	-	-	1963	1965
UCIAMS-281753	*Picea* sp. needle (1)	7.0-7.5	1945	1952	0.981	0.004	-	-	1659	1950*
UCIAMS-281754	*Picea* sp. needle (1)	7.5-8.0	1938	1945	1.2127	0.007	1955	1956	2013	2019
UCIAMS-281755	*Betula* sp. seed (1)	8.0-8.5	1930	1938	0.9748	0.004	-	-	1483	1950*
UCIAMS-281756	*Betula* sp. seed (1)	8.5-9.0	1922	1930	1.1511	0.005	-	-	1665	1950*
UCIAMS-281758	*Betula* sp. seed (1)	9.0-9.5	1914	1922	1.3663	0.006	1959	1962	1984	1986
UCIAMS-281759	*Picea* sp. needle (1)	9.5-10.0	1905	1914	1.1093	0.005	-	-	1640	1950*
